# Editorial: Psychological factors in physical education and sport, volume VI

**DOI:** 10.3389/fpsyg.2026.1937337

**Published:** 2026-08-18

**Authors:** Qing Yi, David Manzano-Sánchez, Giuseppe Battaglia, Carla Chicau Borrego, Manuel Gómez-López

**Affiliations:** 1 College of Physical Education, Dalian University, Dalian, China; 2 Department of Artistic, Musical and Physical Expression, Faculty of Education, University of Murcia, Murcia, Spain; 3 Sport and Exercise Research Unit, Department of Psychology, Educational Sciences and Human Movement, University of Palermo, Palermo, Italy; 4 Sport Sciences School of Rio Maior, Polytechnic Institute of Santarém, SPRINT—Sport Physical Activity and Health Research & Innovation Center, Rio Maior, Portugal; 5 Department of Physical Activity and Sport, Faculty of Sports Sciences, University of Murcia, Murcia, Spain

**Keywords:** mental health, motivation, social support, sport psychology, wellbeing

## Introduction

The interplay between psychological factors and physical activity is a cornerstone of both athletic excellence and public health. In contemporary physical education (PE) and sport, participants ranging from grassroots students to elite athletes face a myriad of cognitive, emotional, and social demands. Understanding how psychological mechanisms drive engagement, mitigate stress, and enhance performance is crucial for developing evidence-based interventions.

The Research Topic “*Psychological Factors in Physical Education and Sport*” was conceptualized to bring together cutting-edge research exploring these dynamics across diverse populations and cultural contexts. Through a rich collection of accepted articles, this Research Topic extends beyond traditional disciplinary boundaries, integrating perspectives from cognitive neuroscience, social psychology, educational theory, and machine learning. This Research Topic synthesizes the contributing research into four distinct yet interconnected themes, providing a comprehensive overview of the current landscape and future directions in sport psychology.

## Cognitive processing, motor competence, and methodological advances

A fundamental component of athletic performance lies in the cognitive processing of temporal, spatial, and visual information. In a compelling narrative review, Sysoeva et al. highlighted how athletes use temporal dilation for subsecond events and compression for suprasecond intervals to optimize decision-making under pressure. This cognitive adaptability is echoed in early childhood development; Yakushina et al. demonstrated that open-skilled sports like football significantly enhance visuospatial working memory in preschool boys compared to martial arts.

The developmental trajectory of actual motor competence (AMC) and its translation into physical activity is heavily mediated by psychological self-perception. Sun G. et al. revealed a critical developmental shift: while physical fitness mediates AMC and activity levels in middle childhood, perceived motor competence becomes the dominant mediator in late childhood. Addressing specific perceptual and cognitive challenges, Zhu G. et al. found that integrating visual tasks into physical exercise not only improved visual acuity but also reduced anxiety in children.

Cultural and technological contexts also substantially influence cognitive appraisals. Kang and Tang identified distinct cross-cultural differences between Korean and Chinese football experts regarding the perceived importance of psychological factors like game intelligence and confidence. Meanwhile, the digital era offers novel inclusion strategies; Kwon et al. illustrated how “high-tech, high-touch” drone soccer programs foster self-efficacy and socially meaningful participation among students traditionally underrepresented in sports. Methodologically, this Research Topic advanced the field by validating robust measurement tools, including the Perceived Physical Literacy Instrument for club coaches (Fu et al.) and the Arabic version of the Engaged Teachers Scale (Boukari et al.). Furthermore, Zhu L. et al. successfully leveraged machine learning algorithms to create highly accurate predictive models of exercise behavior, identifying anxiety and behavioral habits as key predictive factors.

## Mental health, resilience, and wellbeing

The protective role of physical activity against psychological distress was a dominant focal point. Current evidence consistently indicates that physical exercise operates through complex mediating pathways to alleviate anxiety, depression, and burnout.

For vulnerable demographics, such as rural left-behind children, physical activity significantly curbed academic burnout by mitigating loneliness and boosting general self-efficacy (Chen, Zou et al.). Similar protective effects against academic and learning burnout were observed among middle and high school students (Huo et al.). Among university cohorts, exercise reduced depression and psychological distress by enhancing positive mental health (Chen and Zhang), emotional regulation (Wang, J. et al.), and subjective wellbeing (Zhao et al.). Specifically for medical students, high-intensity exercise was found to lower suicidal ideation via improved emotional regulation (Zhang, He et al.).

Psychological resilience emerged as a critical buffer against competitive and academic stressors. Yuan Y. et al. identified latent profiles showing that high resilience neutralizes the negative impacts of anxiety on exercise. Furthermore, interventions like mindfulness training were proven effective in sustainably cultivating resilience in adolescent athletes (Wang and Liu), while martial arts practice enhanced resilience via self-control (Tao and Li). Such resilience is particularly important for referees facing officiating pressure (Feng et al.) and for female college athletes navigating training distress (Wang Z. et al.).

Ultimately, sport participation enhances life satisfaction. This holds true for marathon runners, where recreation specialization enhances life satisfaction through psychological commitment and flow experiences (Xu and Tian), and extends to university students (Gómez-López et al.; Karababa et al.) and even the marital satisfaction of teachers through improved body/sexual self-esteem and spirituality (Snani et al.). Conversely, cognitive-behavioral physical activity acts as a crucial intervention to disrupt ruminative thinking in adolescents (Derelioglu et al.).

## Motivation, emotion, and behavioral regulation

Promoting sustained physical activity participation requires a deep understanding of motivational theories. Grounded in the Control-Value Theory, Li et al. found that a supportive PE environment fosters achievement emotions like enjoyment—while reducing boredom—which in turn solidifies exercise behavior. Similarly, Self-Determination Theory was widely utilized to explain how basic psychological needs differ between individual and team sports (Van Yperen), and how autonomous motivation curbs sports anomie behaviors (Ma et al.).

Grit and self-efficacy are central to this behavioral regulation. Hu Y. et al. detailed how grit enhances exercise through personal growth initiatives, while Chen Y. et al. provided behavioral evidence that a growth mindset drives 800-meter run performance strictly via the “perseverance of effort” dimension of grit. However, structured interventions can produce unexpected results. In a fascinating randomized controlled trial, Aune et al. discovered that a football intervention actually lowered self-reported competence and efficacy, a phenomenon explained by the Dunning–Kruger effect, where improved skill acquisition increased participants' awareness of their own limitations.

In the digital landscape, motivation is increasingly influenced by online environments. While fitness celebrity livestreams promote intentions to engage in physical activity through enhanced social presence and body satisfaction (He et al.), Sun Y. et al. warned of an “S-shaped” curve in fitness gamification, where excessive app features lead to cognitive overload and decreased adherence. Furthermore, mobile phone addiction actively deters physical activity by exacerbating bedtime procrastination (Yu et al.).

## Social dynamics, environmental influences, and inclusion

Sport is inherently social, and the interpersonal environments surrounding athletes strongly influence their psychological outcomes. High-quality coach-athlete relationships directly predict training engagement and skill development (Luo et al.), while supportive teaching styles significantly enhance sports learning engagement (Huang et al.) and ethical orientation (Qiu and Zhang). For teachers themselves, a supportive school environment magnifies the impact of organizational commitment on their professional growth (Hu L. et al.).

The macro-environment, including family and peer support, acts as a vital catalyst. Family educational priorities have evolved across generations to positively influence children's sports participation (Zong et al.). During extreme health crises like the COVID-19 lockdown, family support was indispensable for maintaining active leisure and emotional regulation (García-Gallegos et al.). Peer support also directly enhances mental toughness among adolescent swimmers via self-efficacy (Chen B. et al.). Moreover, perceived social support is essential for encouraging participation in non-traditional sports (Zhang, Wang et al.) and buffering the “dual stress” of academic and athletic demands, particularly through gender-specific pathways (Zhang and Wang).

Addressing the darker social aspects of sport, Erdag et al. explored female athletes' perceptions of sexual harassment across team sports, emphasizing how specific sport cultures dictate exposure and emotional reactions to misconduct. Similarly, Chen X. et al. demonstrated that robust social support networks (athlete–coach–psychologist) mitigate social anxiety and subsequently reduce antisocial behaviors in sports.

Finally, sport participation may have broader spillover effects onto broader societal attitudes. Moravecz et al. and Kraft et al. highlighted how sports events (like the Olympics) and connectedness to nature shape pro-environmental attitudes, eudaimonic wellbeing, and environmental consciousness of Generation Z students and football fans. Meanwhile, Shen et al. mapped the environmental and individual risk factors associated with marathon running, and Shi et al. revealed how water safety knowledge interacts with sensation-seeking to prevent high-risk swimming behaviors. Policy-wise, macro-level school physical education policies effectively translate into active behaviors through students' improved time management and grit (Liu et al.).

## Conclusion and future directions

The diverse contributions within this Research Topic collectively underscore that psychological factors in physical education and sport are not isolated variables, but dynamic, modifiable, and context-sensitive constructs. From the temporal processing of elite athletes to the psychological resilience of rural youth, the findings advocate for holistic, multi-level interventions.

Future research must continue to explore the longitudinal causality of these psychological mechanisms. Furthermore, as digital environments and technology (e.g., social media, e-sports, virtual reality) become increasingly intertwined with physical activity, understanding their psychological impact will be increasingly important. We urge practitioners, coaches, and educators to integrate these empirical insights into pedagogical and training frameworks, fostering environments that are not only competitive but fundamentally supportive of mental health, ethical development, and lifelong wellbeing.

